# 3,4-Methylenedioxymethamphetamine Alters Left Ventricular Function and Activates Nuclear Factor-Kappa B (NF-κB) in a Time and Dose Dependent Manner

**DOI:** 10.3390/ijms11124743

**Published:** 2010-11-26

**Authors:** David A. Tiangco, Sapna Halcomb, Frank A. Lattanzio, Barbara Y. Hargrave

**Affiliations:** 1 Department of Biological Sciences, Old Dominion University, Norfolk, VA 23529, USA; 2 Department of Physiological Sciences, Eastern Virginia Medical School, Norfolk, VA, 23510, USA

**Keywords:** MDMA, ecstasy, NF-kB, nuclear, iNOS, H9c2, cardiac myocytes, rabbit

## Abstract

3,4-Methylenedioxymethamphetamine (MDMA) is an illicit psychoactive drug with cardiovascular effects that have not been fully described. In the current study, we observed the effects of acute MDMA on rabbit left ventricular function. We also observed the effects of MDMA on nuclear factor-kappa B (NF-κB) activity in cultured rat ventricular myocytes (H9c2). In the rabbit, MDMA (2 mg/kg) alone caused a significant increase in heart rate and a significant decrease in the duration of the cardiac cycle. Inhibition of nitric oxide synthase (NOS) by pretreatment with L-NAME (10 mg/kg) alone caused significant dysfunction in heart rate, systolic pressure, diastolic pressure, duration of relaxation, duration of cardiac cycle, and mean left ventricular pressure. Pretreatment with L-NAME followed by treatment with MDMA caused significant dysfunction in additional parameters that were not abnormal upon exposure to either compound in isolation: duration of contraction, inotropy, and pulse pressure. Exposure to 1.0 mM MDMA for 6 h or 2.0 μM MDMA for 12 h caused increased nuclear localization of NF-κB in cultured H9c2 cells. The current results suggest that MDMA is acutely detrimental to heart function and that an intact cardiovascular NOS system is important to help mitigate early sequelae in some functional parameters. The delayed timing of NF-κB activation suggests that this factor may be relevant to MDMA induced cardiomyopathy of later onset.

## 1. Introduction

3,4-Methylenedioxymethamphetamine (MDMA) is a widely used recreational drug among teens and young adults. This substance has molecular similarities to amphetamine and mescaline [1]. Unsurprisingly, users of MDMA report subjective effects attributable to both compounds [1]. Although the physiological impact of this drug has yet to be fully characterized, and exact mechanistic details are still being elucidated, the growing body of evidence continues to support the theory that MDMA abuse is detrimental to the cardiovascular system [1–10].

In humans, MDMA use can cause abnormal increases in heart rate, mean arterial pressure, and myocardial wall stress [2]. In rats, binge pattern intravenous administration of MDMA (9 mg/kg) induces profound and progressive bradycardia with accompanying hypotension [3]. There is evidence that MDMA may directly affect heart function by prolonging Purkinje fiber action potential duration [7]. Other evidence suggests that systemic effects in the form of autonomic dysregulation may be more important in the rhythmic and pressor abnormalities seen with MDMA abuse [4,10]. This may involve increased net release of norepinephrine and alpha-adrenergic receptor interaction [1,4,5,9]. In addition, abnormally elevated intracellular calcium, as observed in our own previous experiments with MDMA, may lead to further complications including myocardial hypertrophy, fibrosis, and apoptosis [8,11,12].

The direct mechanism(s) by which MDMA causes adverse cardiovascular effects is still under investigation. In cardiac myocytes, one important transcription factor activated by MDMA is nuclear factor-kappa B (NF-κB) [11]. NF-κB is ubiquitously found in a wide variety of cell types as a heterodimer consisting of p50 and p65 subunits [13–16]. Under normal conditions, NF-κB is localized mainly in the cytoplasm where it is inhibited by the I-kappa B (IκB) family of regulatory proteins [17]. Activation occurs when IκB becomes phosphorylated, ubiquitinated, and subsequently degraded allowing for translocation into the nucleus where NF-κB binds to κB-response elements in the promoter/enhancer regions of various genes [13,18–23]. This process upregulates the expression of various downstream inflammatory factors including cytokines, chemotactic proteins, and cellular adhesion molecules [23]. There are several well known inducers of NF-κB including interleukin-1β (IL-1β), interleukin-6 (IL-6), interleukin-8 (IL-8), tumor necrosis factor-α (TNF-α), lipopolysaccharide (LPS), physical trauma, ionizing radiation, and free radicals [24–28]. Previous studies in our laboratory indicate that illicit drugs such as cocaine can also activate this transcription factor [11,29].

In the heart, NF-κB activation is closely associated with pro-inflammatory as well as anti-apoptotic conditions. Rat cardiac myocytes in culture exposed to an oxidizing environment, such as co-incubation with hydrogen peroxide, show an increased NF-κB activity [30]. Myocardial intercellular adhesion molecule-1 (ICAM-1) and vascular cell adhesion molecule-1 (VCAM-1) gene expression are effectively reduced when NF-κB is inhibited [31,32]. These cell surface proteins are important in mediating inflammatory cell recruitment and infiltration by acting as counter-receptors for circulating monocyte and lymphocyte integrins [32]. The anti-apoptotic nature of NF-κB has been demonstrated in primary cultures of rat ventricular myocytes. In these cells, TNF-α induced apoptosis is significantly enhanced by NF-κB inhibition [33].

Earlier research reveals important information concerning the effects of MDMA on redox balance within the cell, a critical aspect in NF-κB regulation. MDMA has been shown to simultaneously increase reactive oxygen species (ROS) production and deplete reduced glutathione levels in hepatic stellate cells [34]. MDMA also increases hydroxyl radical formation in the rat brain [35]. Theoretically, the oxidative stress created by MDMA in these studies can promote NF-κB activation, modulate downstream genetic expression, and induce an inflammatory response. Supporting evidence exists in rats given binge-pattern administration of MDMA over the course of several weeks [3,36]. These animals show histological signs of increased lymphocyte infiltration and inflammatory damage to the myocardium [3]. They also display significantly elevated nitrotyrosine content of proteins (an index of ROS activity) isolated from the left ventricle [36].

We have reported that NF-κB is activated by MDMA in a dose-dependent manner [11]. By way of genetic array technology, we have also observed that several NF-κB dependent genes are upregulated at the transcriptional level. Some of these genes, such as PCNA, Cox-2, and VCAM-1 have been implicated in cardiac disease states such as heart failure, hyperplasia, fibrosis and inflammation [32,37,38]. Others, such as Gadd45 and RAD23A, are involved with survival and compensatory responses [39,40]. While genes, such as inducible nitric oxide synthase (iNOS) and brain natriuretic peptide (BNP) are upregulated in heart failure, but were not significantly modulated in response to MDMA under our conditions. It is possible that iNOS and BNP were affected at earlier time points [41,42].

iNOS is an important signaling molecule in the cardiovascular system and is under NF-κB control [41]. The metabolic end product of the enzyme - nitric oxide (NO) is a well known vasodilator and helps to regulate blood pressure [43–45]. In the compromised endothelium, as commonly seen in atherosclerosis, NO output is significantly diminished [46,47]. Atherosclerosis has aberrant functional consequences for the heart such as increased vasoconstriction and elevations in heart rate and contractility (inotropy) that may be exacerbated by MDMA use.

The full scope of MDMA induced effects on the cardiovascular system and the extent of NF-κB involvement remains incompletely characterized. Therefore, the purpose of the current study was to observe the cardiac functional response to acute MDMA administration in the intact rabbit and to further characterize molecular events in cultured ventricular myocytes as a result of direct MDMA exposure. We also observed the effects of MDMA on nitric oxide synthase inhibition in the cardiovascular system.

## 2. Materials and Methods

### 2.1. *In Vivo* Experiments

Sixteen adult New Zealand White (NZW) rabbits (*Oryctolagus cuniculus*) were randomly divided into 3 treatment groups: Placebo (n = 5), MDMA (n = 7), and L-NAME + MDMA (n = 4). A Digi-Med Heart Performance Analyzer-τ model 410 (Micro-Med; Tustin, CA) was used to measure left ventricular systolic pressure (MaxP), diastolic pressure (EDP), inotropy (dP/dt), lusitropy (NdP/dt), duration of contraction (DCON), duration of 1/2 relaxation (1/2R), and heart rate (HR). From these parameters, three additional parameters were calculated: duration of cardiac cycle (CCycle), mean ventricular pressure (MeanP), and pulse pressure (PulseP). The analyzer was connected to a Millar model SPR-524 catheter (Millar Instruments, Inc.) which contains a specialized sensor tip designed for transducing mechanical signals directly from within the left ventricular chamber into electrical signals that can be interpreted by the analyzer and recorded by the computer.

Animals were placed under a surgical plane of anesthesia by administering ketamine (40 mg/kg) and xylazine (5 mg/kg) intramuscularly. A 2 cm longitudinal mid-line incision was made parallel to the trachea. The left common carotid artery was carefully isolated from the surrounding tissues and a Millar catheter gently inserted into the left ventricle. A 24 G ½ inch catheter was inserted into the marginal ear vein of the rabbit for the injection of all drugs and solutions. Animals in the placebo group were given 0.9% sterile isotonic saline intravenously (IV). Animals in the MDMA group received 2 mg/kg MDMA IV bolus. Animals in the L-NAME + MDMA group were pretreated with 10 mg/kg L-NAME (non-specific NOS inhibitor) 10 min before 2 mg/kg MDMA was injected, IV. Left ventricular functional data was recorded every 10 s by the computer during the entire experiment. After data collection, the animal was euthanized and the heart was carefully removed and weighed on an analytical balance. The left ventricle was separated from all other tissue, snap-frozen with liquid nitrogen, and stored at −80 °C until analysis. All functional data were normalized to baseline/pre-injection parameters and statistically analyzed as ratiometric units using single-factor ANOVA for each time point. The functional effects of L-NAME alone relative to pre-injection were analyzed via paired t-test.

### 2.2. *In Vitro* Experiments

#### 2.2.1. Reactive Oxygen Species Assay

These experiments were designed to measure the effects of MDMA on reactive oxygen species (ROS) generation in cultured cardiac myocytes. H9c2 cells (*Rattus norvegicus*) were grown to 85–90% confluency in T-75 flasks at 37 °C and 5% atmospheric CO_2_ (incubator controlled). Culture media consisted of 15 mL Dulbecco’s Modified Eagle’s Medium™ containing 10% fetal bovine serum (FBS). Cells and media were purchased from American Type Culture Collection (ATCC; Manassas, VA, USA). Cell manipulations were carried out under a tissue culture hood using aseptic technique. The green fluorescent indicator 2′,7′-dichlorofluorescin diacetate (Molecular Probes) was used to measure the presence of MDMA induced ROS generation. On day 1, with the aid of a hemacytometer, approximately 150,000 H9c2 cells (approximately 1.05 × 10^5^ cells/cm^2^) were plated onto a sterile multi-well culture plate (Corning). The cells were then returned to the incubator for 24 hours.

On day 2, the overlying media was removed and discarded. The cells were washed with 1 mL of Hank’s Media (ATCC) in order to remove residual cell culture media which contained a red coloring that might interfere with the indicator. Afterward, 1 mL of fresh Hank’s Media was placed in each well. 2′,7′-dichlorofluorescin diacetate was dissolved in dimethyl sulfoxide (DMSO) (Sigma). To load the cells, 1 mL of the 5% indicator solution was added to each well and the culture plate was gently swirled for 1 min. The culture plate was then wrapped in aluminum foil and returned to the incubator for 30 min.

Following the loading period, the cells were washed twice with 1 mL of Hank’s Media. Next, 2 mL of fresh Hank’s Media was placed into each well and the plate was returned to the incubator for 30 min prior to drug administration. The following concentrations of MDMA were tested: 1 × 10^−4^ M, 1 × 10^−3^ M, and 1 × 10^−2^ M. The drug was dissolved in Hank’s Media and 2 mL of each concentration was gently added to the appropriate wells. Each well served as its own 0 M control. The cells were imaged using a Zeiss model 510 LSM confocal microscope (Carl Zeiss) equipped with a variable wavelength argon laser, epifluorescent filters, and a digital camera. Cells generating ROS exhibited a quantifiable green fluorescence. Images were captured 2 and 5 min after drug exposure and then analyzed by the program Metamorph (Universal Imaging) for densitometry. The results were exported to Excel (Microsoft) and statistically analyzed using paired t-test.

#### 2.2.2. ELISA for NF-κB

These experiments were designed to determine the effect of MDMA on the degree of NF-κB activation in cultured cardiac myocytes. H9c2 cells were grown and maintained as previously described in our ROS experiments. On day 1 of the experiment, approximately 1 × 10^6^ cells were plated onto each well of a sterile 6-well culture plate (approximately 1.05 × 10^5^ cells/cm^2^) and returned to the incubator for 24 hours. On day 2, the cells were stimulated with MDMA or placebo (culture media). Two concentrations of MDMA were used: 1.0 mM or 2.0 μM. To stimulate the cells, the drug was dissolved in an appropriate volume of cell culture media and 5 mL of total solution added to each well. An equal volume of cell culture media (without drug) was added to cells that served as our paired controls (0 mM MDMA). The cells were then returned to the incubator for either 3, 6, or 12 h. These concentrations and exposure times were specifically chosen because of evidence in human subjects suggesting that peak MDMA plasma levels occur between 2–4 h after drug administration [48]. Each time point and drug concentration was tested independently in separate experiments.

At the end of the drug exposure period, nuclear extracts were obtained per manufacturer instructions using an extraction kit (AY2002) from Panomics (Fremont, CA). Briefly, the cells were washed twice with 10 mL of isotonic phosphate buffered saline (PBS), pH 7.4. Cells were lysed using a lysis buffer (1 mL solution of 10 mM HEPES, 10 mM KCl, 10 mM EDTA; 10 μL of 100 mM DTT; 10 μL protease inhibitor cocktail; 40 μL of 10% IGEPAL) for 10 min. The cells in each well were then scraped and transferred to a separate 1.5 mL tube and centrifuged at 14,000 × g for 3 min at 4 °C. The pellets were retained and placed on ice.

A separation buffer (147 μL solution of 20 mM HEPES, 0.4 M NaCl, 1 mM EDTA, 50% glycerol; 1.5 μL protease inhibitor cocktail; 1.5 μL of 100 mM DTT) was used to isolate nuclear proteins. The pellets were resuspended in the separation buffer and gently agitated on ice for 2 h. The nuclear extracts were stored at −80 °C in sterile nuclease-free microcentrifuge tubes until analysis. A quantitative assay (Bradford) was used to determine the total protein concentration of each sample.

To measure the presence of activated NF-κB in the nuclear extracts, a modified ELISA based kit (EK1010) from Panomics (Fremont, CA) was used. A specialized 96 well microplate pre-coated with NF-κB binding consensus oligonucleotide was incubated with 2.0 μg of total nuclear extract. Primary antibody to the p50 subunit of NF-κB was added to each well at a dilution of 1:100. Conjugated secondary antibody was added to each well at a dilution of 1:1000. Colorimetric substrate was added and the reaction allowed to incubate at room temperature for 15 min. The plate was then analyzed at A450 nm using a FluoStar™ spectrophotometric microplate reader (BMG Labtech, Inc. Durham, NC). The results were exported to Excel (Microsoft) and statistically analyzed using the paired t-test.

In order to test for NF-κB activity in left ventricular heart tissues of acutely treated rabbits, another set of experiments was performed. Nuclear extracts were obtained per manufacturer instructions using an extraction kit (AY2002) from Panomics (Fremont, CA). A quantitative assay (Bradford) was used to determine the total protein concentration of each sample.

To measure the presence of activated NF-κB in the nuclear extracts, a modified ELISA based kit (P/N 12490) from Panomics (Fremont, CA) was used. A specialized 96 well microplate pre-coated with streptavidin was incubated with a mixture containing 10.0 μL binding buffer, 2.5 μL NF-κB specific biotinylated oligonucleotide probe, and 1.0 μg of total nuclear extract. Primary antibody to the p50 subunit of NF-κB was added to each well at a dilution of 1:200. Conjugated secondary antibody was added to each well at a dilution of 1:200. Colorimetric substrate was added and the reaction was allowed to incubate at room temperature for 15 min. The plate was then analyzed as previously described. The results were exported to Excel (Microsoft) and statistically analyzed using the two-sample t-test.

## 3. Results

### 3.1. *In Vivo* Experiments

**Figure 1.**
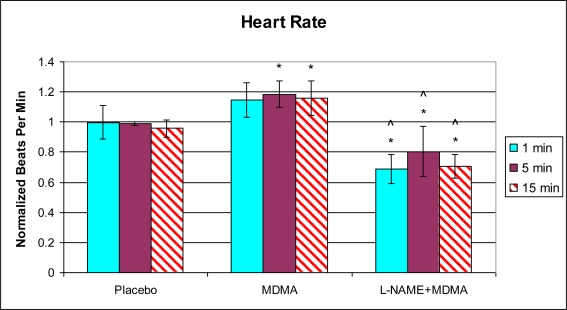
Normalized heart rate (mean ± SD). Placebo group (n = 5) received sterile isotonic saline. MDMA group (n = 7) received 2 mg/kg MDMA. L-NAME + MDMA group (n = 4) received 10 mg/kg L-NAME for 10 min followed by 2 mg/kg MDMA. * p < 0.05 compared to Placebo. ^ p < 0.05 compared to MDMA.

**Figure 2.**
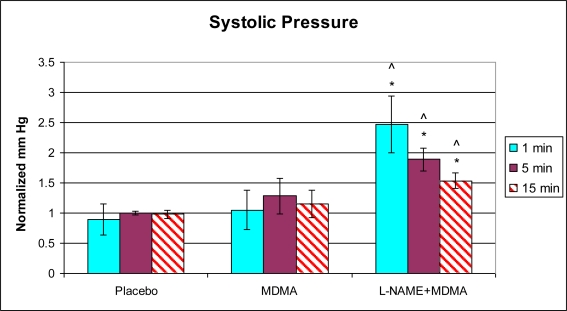
Normalized systolic pressure (mean ± SD). Placebo group (n = 5) received sterile isotonic saline. MDMA group (n = 7) received 2 mg/kg MDMA. L-NAME + MDMA group (n = 4) received 10 mg/kg L-NAME for 10 minutes followed by 2 mg/kg MDMA. * p < 0.05 compared to Placebo. ^ p < 0.05 compared to MDMA.

Analysis of left ventricular mechanical function revealed that MDMA alone significantly increased heart rate 5 min after injection (Figure 1) and decreased the duration of the cardiac cycle at the 15 min time point (Figure 2). The elevation in heart rate persisted throughout the remainder of post-injection monitoring. No significant changes in any of the other functional parameters were observed in the MDMA treated group relative to the Placebo group.

**Figure 3.**
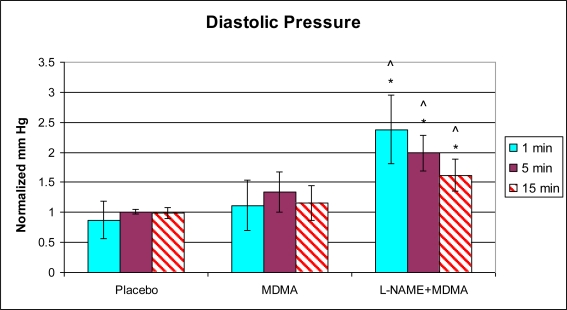
Normalized diastolic pressure (mean ± SD). Placebo group (n = 5) received sterile isotonic saline. MDMA group (n = 7) received 2 mg/kg MDMA. L-NAME + MDMA group (n = 4) received 10 mg/kg L-NAME for 10 minutes followed by 2 mg/kg MDMA. * p < 0.05 compared to Placebo. ^ p < 0.05 compared to MDMA.

**Figure 4.**
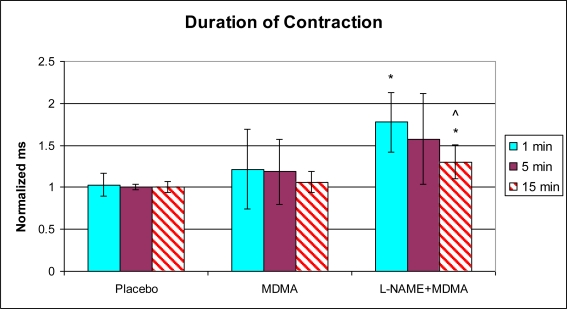
Normalized duration of contraction (mean ± SD). Placebo group (n = 5) received sterile isotonic saline. MDMA group (n = 7) received 2 mg/kg MDMA. L-NAME + MDMA group (n = 4) received 10 mg/kg L-NAME for 10 minutes followed by 2 mg/kg MDMA. * p < 0.05 compared to Placebo. ^ p < 0.05 compared to MDMA.

**Figure 5.**
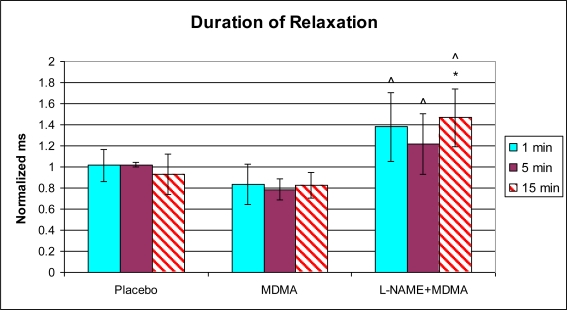
Normalized duration of relaxation (mean ± SD). Placebo group (n = 5) received sterile isotonic saline. MDMA group (n = 7) received 2 mg/kg MDMA. L-NAME + MDMA group (n = 4) received 10 mg/kg L-NAME for 10 minutes followed by 2 mg/kg MDMA. * p < 0.05 compared to Placebo. ^ p < 0.05 compared to MDMA.

**Figure 6.**
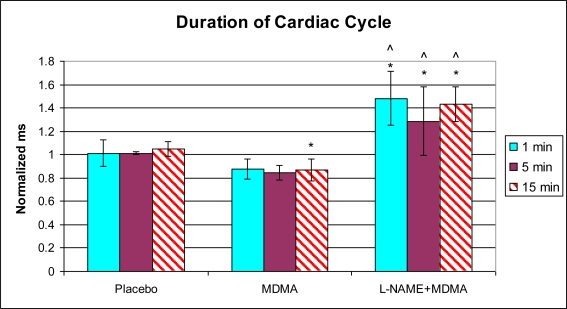
Normalized duration of cardiac cycle (mean ± SD). Placebo group (n = 5) received sterile isotonic saline. MDMA group (n = 7) received 2 mg/kg MDMA. L-NAME + MDMA group (n = 4) received 10 mg/kg L-NAME for 10 min followed by 2 mg/kg MDMA. * p < 0.05 compared to Placebo. ^ p < 0.05 compared to MDMA.

**Figure 7.**
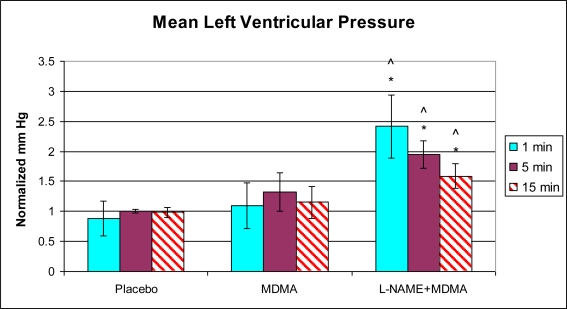
Normalized mean pressure (mean ± SD). Placebo group (n = 5) received sterile isotonic saline. MDMA group (n = 7) received 2 mg/kg MDMA. L-NAME + MDMA group (n = 4) received 10 mg/kg L-NAME for 10 minutes followed by 2 mg/kg MDMA. * p < 0.05 compared to Placebo. ^ p < 0.05 compared to MDMA.

**Figure 8.**
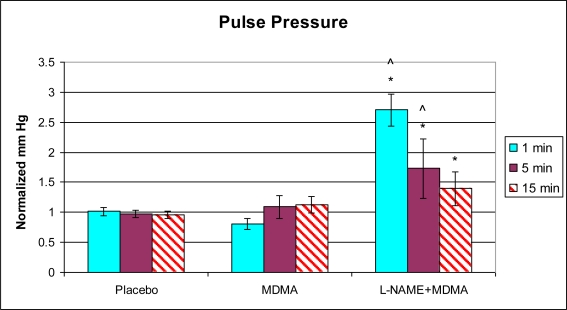
Normalized pulse pressure (mean ± SD). Placebo group (n = 5) received sterile isotonic saline. MDMA group (n = 7) received 2 mg/kg MDMA. L-NAME + MDMA group (n = 4) received 10 mg/kg L-NAME for 10 minutes followed by 2 mg/kg MDMA. * p < 0.05 compared to Placebo. ^ p < 0.05 compared to MDMA.

**Figure 9.**
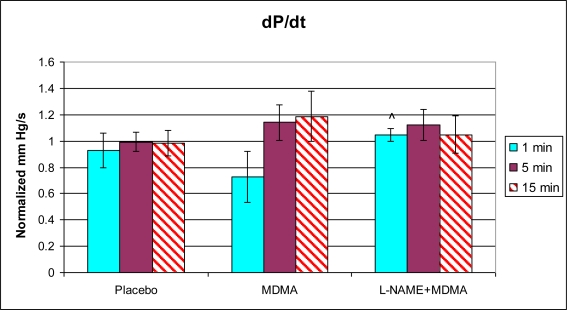
Normalized dP/dt (mean ± SD). Placebo group (n = 5) received sterile isotonic saline. MDMA group (n = 7) received 2 mg/kg MDMA. L-NAME + MDMA group (n = 4) received 10 mg/kg L-NAME for 10 minutes followed by 2 mg/kg MDMA. ^ p < 0.05 compared to MDMA.

In the group of animals pretreated with L-NAME and then given MDMA (L-NAME + MDMA), heart rate (Figure 1) was significantly decreased while systolic and diastolic pressures, duration of contraction, duration of relaxation, duration of cardiac cycle, mean pressure, and pulse pressure were all significantly elevated from baseline (Figures 2–9). dP/dt showed a significant increase relative to the MDMA group at the 1 min mark.

**Figure 10.**
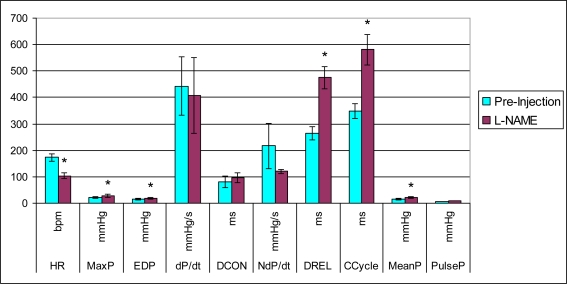
Effects of L-NAME on cardiac functional parameters (mean ± SD). L-NAME + MDMA group (n = 4). Pre-injection parameters were record for 15 minutes. Post-injection of 10 mg/kg L-NAME was recorded for 10 minutes. * p < 0.05 compared to pre-injection.

L-NAME alone significantly decreased heart rate, but elevated systolic pressure, diastolic pressure, duration of relaxation, duration of cardiac cycle, and mean pressure (Figure 10). DCON, dP/dt, and PulseP were not significantly affected by L-NAME alone. However, after the addition of MDMA, these parameters were significantly increased.

### 3.2. *In Vitro* Experiments

#### 3.2.1. Reactive Oxygen Species Assay

**Figure 11.**
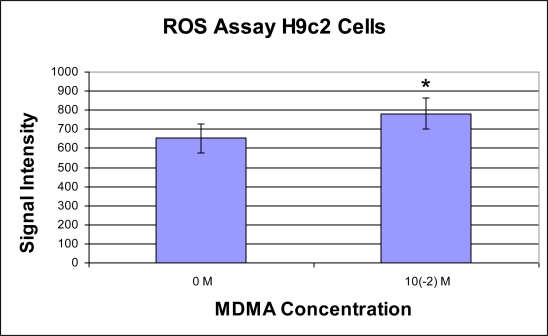
ROS assay signal intensity at 5 min (mean ± SD). Each well served as its own control (n = 3). * p < 0.05 compared to Control Signal.

Using cultured H9c2 cells, we observed a significant increase in ROS generation in response to MDMA exposure. ROS generation was significantly elevated relative to 0 M control 5 min after exposure to 1 × 10^−2^ M MDMA (Figure 11).

#### 3.2.2. ELISA for NF-κB

**Figure 12.**
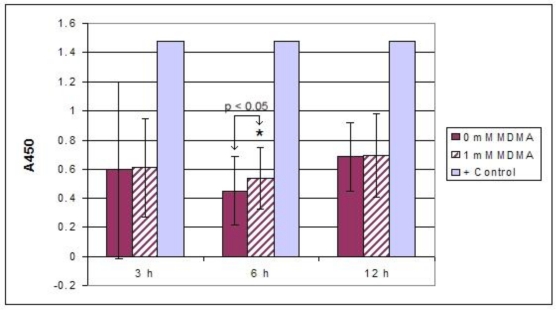
ELISA for NF-κB nuclear localization (mean ± SD). Either 0 mM MDMA (media only) (n = 3) or 1 mM MDMA (n = 3) was used at each time point. Each experiment was performed independently. A kit-provided assay-level positive control (+Control) of nuclear extracts prepared from IL-1β treated HeLa cells was included during the plate run. * p < 0.05.

**Figure 13.**
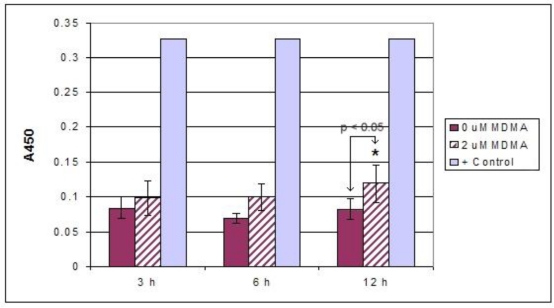
ELISA for NF-κB nuclear localization (mean ± SD). Either 0 μM MDMA (media only) (n = 3) or 2 μM MDMA (n = 3) was used at each time point. Each experiment was performed independently. A kit-provided assay-level positive control (+Control) of nuclear extracts prepared from IL-1β treated HeLa cells was included during the plate run. * p < 0.05.

We observed a significant increase in nuclear localization of NF-κB in H9c2 cells exposed to 1.0 mM MDMA at the 6 h time point (Figure 12). Measurements taken at the 3 h and 12 h time points for cells exposed to 1 mM MDMA were not significantly different from their paired controls. We also observed significant nuclear localization of NF-κB with the 2.0 μM dose, however this occurred at the 12 h time point (Figure 13). In addition, we did not observe a significant difference in NF-κB activity between the placebo and MDMA groups in myocardial tissue specimens from our functional experiments (data not shown).

## 4. Discussion

### 4.1. Effects of MDMA

In this study we further characterize the effects of MDMA on the cardiovascular system. The widespread abuse of MDMA, along with the possibility of direct and indirect effects contributing to both acute and long-term sequelae, makes this drug particularly important in terms of potential cardiotoxicity. We show, in the intact rabbit, that MDMA can cause acute functional abnormalities of the left ventricle and that NOS signaling may be an important protective response. We also show, in cultured cardiac myocytes, that MDMA has the potential to activate NF-κB, a key molecular transcription factor associated with myocardial pathology. The current findings support earlier reports that implicate MDMA in augmenting both sympathetic responses and NF-κB activity [1–5,9,10].

Compared to placebo, MDMA alone caused a 21% increase in heart rate 15 min after injection and significantly decreased the duration of the cardiac cycle at the same time point (17%). Due to the early onset of the cardiovascular responses, and the fact that NF-κB activation in our cultured cardiac myocytes was not observed at or before the 3 h time point with either MDMA dose, these responses may not be the result of interaction with the myocardial NF-κB pathway. Two possibilities may explain these results. The injected MDMA may have directly and rapidly affected cardiovascular calcium homeostasis in blood vessels and led to the slight increase in systolic and diastolic pressures. This is unlikely, however, because the changes in pressures were slight and non-significant. It is possible that MDMA may have stimulated systemic sympathetic activity by causing a net increase in epinephrine and norepinephrine release from central stores and/or peripheral nerve endings. This idea is supported by the fact that there was a significant increase in heart rate 5 and 15 min after MDMA injection and suggests a possible direct effect of MDMA on the sinoatrial node of the heart or indirect stimulation via catecholamines.

Previously, we have demonstrated that MDMA causes a significant increase in intracellular calcium in cultured H9c2 cells within a few minutes of drug administration [11]. Elevated calcium levels may affect intracellular signaling pathways leading to diverse pathological responses including fibrosis and hypertrophy. Disruptions in calcium homeostasis may also alter myocardial excitability and contractility, which may induce ventricular arrhythmia and other more immediate functional abnormalities such as the increased heart rate and decreased cardiac cycle duration observed in the present study. An increase in heart rate associated with a decrease in the duration of the cardiac cycle may suggest that filling and ejection of blood in the heart exposed to MDMA may be acutely compromised.

In cultured H9c2 heart cells, a significant increase in ROS generation was observed 5 min after 1 × 10^−2^ M MDMA exposure. Although we exposed the H9c2 heart cells to MDMA acutely and measured ROS generation 5 min later, the half-life of MDMA has been reported to be approximately 9 ± 2.2 h [49]. In addition, there is evidence that MDMA metabolites adversely affect cardiac myocyte redox balance [6]. MDMA is metabolized into toxic metabolites including HMMA, MDA, and HMA, and may lead to the formation of peroxinitrite, all of which may prolong and exacerbate the deleterious effects of the drug [50–52]. *In vivo* and *in vitro* studies provide evidence supporting the role of both excessive monoamine release and oxidative stress in cardiovascular conditions (such as ischemia-reperfusion injury, hypertension, catecholamine-induced cardiomyopathy, and heart failure) that lead to cardiovascular dysfunction [53–55]. In addition to generation of ROS, 1.0 mM of MDMA also significantly increased the activity of NF-κB in H9c2 heart cells in culture after 6 h of exposure. After 12 h of exposure, 2.0 μM MDMA significantly enhanced NF-κB activity. This redox sensitive transcription factor is known to upregulate the expression of genes associated with myocardial inflammation, hypertrophy, and fibrotic scar formation.

Preliminary evidence from our lab indicates that NF-κB is not significantly activated in H9c2 cells exposed to MDMA for less than 3 h (data not shown). It is possible that under the current experimental conditions 3 h is not sufficient time for significant nuclear accumulation of this transcription factor. Based upon our previous investigation [11], a biphasic response to 1.0 mM MDMA is suggested since sometime after 6 h, nuclear presence of NF-κB becomes significantly degraded, but then reappears at the 24 h time point according to our genetic reporter study. It is uncertain why this drug is associated with a temporal response with regard to NF-κB. It is possible that some metabolic by-product of MDMA may be participating later in the NF-κB response and/or cellular adaptation is occurring in response to the persistent presence of metabolites over extended periods of time.

Significant NF-κB activity was absent in the myocardial tissue samples from our functional experiments. Since the animals were exposed to MDMA for only 15 min prior to euthanasia and tissue harvesting, the lack of NF-κB activity is in agreement with our cell culture findings within the same timeframe. Therefore, it is unlikely that any of the acute functional responses induced by MDMA are due to the activation of myocardial NF-κB and the consequent expression of downstream dependent genes such as inflammatory cytokines and adhesion molecules. It is more likely that other tissues, such as the vasculature and/or sympathetic peripheral nerve endings may be responsive to this drug within the tested timeframe and may contribute to the abnormal functional findings in the left ventricle by increasing peripheral resistance. Another possibility is that MDMA induced ROS generation, as observed in the current study, and abnormal intracellular Ca^2+^ elevation, as observed in our previous study [11], presents a more immediate threat to normal cardiac function since these events were precipitated rapidly after drug exposure.

### 4.2. MDMA and L-NAME

Since MDMA activates NF-κB, we predicted that downstream dependent genes such as inducible nitric oxide synthase (iNOS) would be upregulated as a result of MDMA exposure. iNOS is a cardioprotective enzyme closely associated with compensatory actions in the heart during ischemic preconditioning and inflammatory cytokine exposure [56,57]. It is possible that MDMA stresses the heart and causes the myocardium to respond in an adaptive manner by increasing the synthesis of this enzyme. In the current study, we performed an independent series of *in vitro* experiments in which we again exposed H9c2 heart cells in culture to 2.0 μM and 1.0 mM concentrations of MDMA. We did not observe a significant change in the expression of iNOS at the protein level. Since iNOS expression in cardiac myocytes is NF-κB dependent, it is still possible that a certain degree of iNOS expression occurred below the threshold for detection, or at time points earlier than we examined.

The importance of an intact nitric oxide signaling system in reducing the adverse functional effects of MDMA is readily apparent in our functional experiments. L-NAME is a well characterized inhibitor of the major NOS isoforms [58,59]. In the rat, this compound induces a dose-dependent increase in heart rate and mean arterial pressure [58]. L-NAME also decreases blood flow by increasing vasoconstriction in several vascular beds including those associated with the kidneys, mesenteries, and hindquarters [60]. Endothelial NOS (eNOS) is particularly important to normal cardiovascular function. In mice, decreased expression of eNOS reduces vasorelaxation capacity and increases arterial pressure [61].

In our experiments, injection of L-NAME alone caused a significant increase in heart rate, left ventricular systolic and diastolic pressures, the duration of left ventricular relaxation and the duration of the cardiac cycle. When the animal was given MDMA alone, heart rate increased and the duration of the cardiac cycle decreased. These results are consistent with what was expected since the duration of the cardiac cycle is inversely proportional to the heart rate. In the presence of L-NAME, MDMA decreased heart rate and increased the duration of the cardiac cycle. These results may suggest that one mechanism used by MDMA to alter heart function is via the nitric oxide synthase pathway. Nitric oxide has been shown to play a role in the modulation of sympathetic nerve activity and baroreflex sensitivity [62]. For instance, if MDMA and L-NAME are both sympathomimetic synergists, then the increased systolic pressure induced by L-NAME may be enhanced by the addition of MDMA. The consequent decrease in heart rate may be exclusively due to the effects of L-NAME or the compensatory baroreceptor reflexes of the intact cardiovascular system responding to the slight change in pressure. It is therefore not possible, in this model, to separate the reflex component of the response to these drugs.

The use of MDMA may have important implications for individuals with a compromised vascular endothelium, as seen in atherosclerosis. Endothelial cells are an important source of nitric oxide which helps to maintain normal blood pressure by regulating vascular diameters and peripheral resistance. Therefore, users of MDMA with extensive atherosclerotic lesions may be at increased risk for the development of acute rhythmic abnormalities and subsequent heart failure.

## 5. Conclusions

This study provides evidence that MDMA is acutely detrimental to normal heart function. The current evidence also suggests that an intact cardiovascular NOS system exerts a partially protective response to the drug in some, but not all, functional parameters. It is likely that MDMA induced autonomic dysregulation contributes to the functional abnormalities we observed and that these effects are characteristic of the immediate/early consequences of abuse. In addition, MDMA may be toxic to the myocardium due to its ability to increase ROS generation and activate NF-κB. However, since the NF-κB response was significantly elevated *in vitro* only after several hours past initial exposure and not elevated *in vivo* during the timeframe of our functional studies, the acute detrimental effects of MDMA on heart function may not be related to myocardial activation of this transcription factor. Nevertheless, it remains possible that sequelae of latter onset may be caused by NF-κB activation and the expression of genes under its control. Further investigations involving MDMA and the effects of various patterns and durations of drug exposure will yield important additional information as to the true nature of this substance and its impact on the cardiovascular system.
